# Sentiment analysis of cancer screening in Chinese social media: Qualitative studies based on machine learning

**DOI:** 10.1371/journal.pone.0336119

**Published:** 2025-11-05

**Authors:** Qi Zhou, Lingling Qian, Luyu Wu, Haiqian Wu, Junwei Ye, Qinrou Yu, Xiangnan Gu, Yueli Zhu

**Affiliations:** 1 Integrated Traditional and Western Medicine Hospital of Linping District, Hangzhou, Zhejiang Province, China; 2 Tongji zhejiang college, Huzhou, Zhejiang Province, China; 3 Fengyang College, Shanxi Medical University, Fengyang, Shanxi Province, China; 4 HuZhou Normal University, Hangzhou, Zhejiang Province, China; 5 Nursing department, Hangzhou normal university, Hangzhou, Zhejiang Province, China; 6 Zhejiang University, Hangzhou, Zhejiang Province, China; Xiangya Hospital Central South University, CHINA

## Abstract

**Purpose:**

Explore public perceptions and sentiments about cancer screening on social media. The dissemination of misinformation and negative attitudes continue to impede the access of many individuals with perceived risk to cancer screening services despite their awareness of the necessity and concept of early cancer screening.

**Methods:**

This study was divided into five steps: data collection, data cleaning, data standardization, sentiment analysis, and content analysis.

**Results:**

This study analyzed 796 social media comments (53,151 words) from Weibo, Zhihu, and Xiaohongshu to explore public sentiments toward cancer screening. Seven emotion categories emerged: good, happy, surprise, anger, disgust, fear, and sadness. Positive emotions reflected trust in physicians, financial support, and perceived screening effectiveness, whereas negative emotions reflected fear of cancer, stigma, and procrastination.

**Conclusion:**

The findings of this study include the development of health communication strategies, the promotion of public screening participation, and the improvement of nursing personalization and emotional sensitivity. These findings highlight barriers and facilitators for cancer screening promotion in China and inform targeted nursing communication strategies.

**What is already known**

Cancer screening enhances cure rates, decreases treatment costs, preserves quality of life, raises health awareness, and promotes healthy lifestyles.

Social media can increase cancer screening acceptance rates. However, the researchers ignored comments about cancer screening that reflected individuals’ perceptions and sentiments in social media.

**What this paper adds**

The results of positive emotions showed positive psychological drive, trust, financial support, and screening effectiveness. The results of happy emotions showed resilience and safety. The results of surprise emotions showed self-doubt.

The results of fear emotions showed a lack of Knowledge about cancer and its screening methods and a high-risk perception, which can lead to avoidance of screening. The results of sad emotions showed procrastination and low perceived susceptibility, the regret of anticipated death, loss, loneliness, and trauma.

## 1. Introduction

Cancer is a leading cause of morbidity and mortality worldwide, with lung, colorectal, and breast cancers among the most common [1]. Prevention and early detection are essential to reducing incidence and mortality [2,3]. Cancer screening enhances cure rates, decreases treatment costs, preserves quality of life, and promotes health awareness and healthy lifestyles [2].

The dissemination of misinformation and negative attitudes continue to impede the access of many individuals with perceived risk to cancer screening services despite their awareness of the necessity and concept of early cancer screening [2]. Specifically, individuals often fail to comply with screening recommendations due to their lack of Knowledge, personal health beliefs, low self-efficacy, or lack of drive [4–6]. The test acceptance rates for cervical, colorectal, and breast cancers are declining [3]. Consequently, we must take the necessary measures to increase the acceptance of screening [2,7,8].

Social media increases the acceptance rates of cancer screening [9–11]. For caregivers, social media allows nurses to use their influence as opinion leaders to promote health education and cancer screening [12]. For the public, Social media provides a plethora of information about the importance of cancer screening, the risks and benefits, and various screening methods [13]. Many cancer patients and previous screening participants will use social media to discuss their firsthand experiences and feelings. It helps others comprehend the screening procedure and results and alleviates their fears. However, the researchers neglected comments representing user perceptions and sentiments on cancer screening [14,15].

Public attitudes heavily influence the decision to use cancer screening services [16–18]. Attitudes reflect the public’s perception of health hazards and preventive measures [19,20]. The participation and acceptability of cancer screening may be enhanced when the general public recognizes the benefits or is influenced by others. Unfortunately, the research on sentiment analysis for public cancer screening is limited. It focuses on only a few types of cancer and lacks in-depth sentiment analysis [21]. The study is primarily focused on particular types, such as breast and colon cancer, and various treatment techniques [22]. There is an urgent need for comprehensive cancer screening services that can evaluate individual screening needs, optimize resource allocation, and increase public acceptance and participation, highlighting the importance of this issue to the reader. In addition, most studies are limited to acquiring positive or negative results, with a need for further analysis of the causes and attitudes [16,23]. Consequently, this situation hampers the applicability of the findings to health education. While prior studies examined public sentiment toward specific cancer types (e.g., breast, colorectal), few have systematically analyzed emotions surrounding cancer screening more broadly in the Chinese social media context. Cultural, linguistic, and healthcare system differences may shape public perceptions uniquely, highlighting the need for this study.

Certain disadvantages exist when a researcher utilizes traditional methods, including questionnaires and interviews, to acquire the public’s affective attitudes [2]. Interviews make it difficult for the researcher to obtain honest opinions because the researcher fears offending if negative attitudes are expressed. Questionnaires can be costly, and the survey’s specific questions limit the range of responses [22]. Therefore, analyses based on social media comments may overcome the limitations of traditional survey methods and give researchers a fresh perspective for future studies. The potential of social media for improving healthcare research is a reason for optimism and hope in the field.

According to the literature, cancer screening research has primarily been based on the English-language Internet, such as Twitter and YouTube [22,23]. Thus, researchers and medical professionals must focus on research studies regarding Internet cancer screening in China. The large number of cancer patients in China and disparities in culture, health policies, and healthcare systems can influence the implementation, promotion, and acceptance of cancer screening [15,20].

In conclusion, this study addresses these gaps by analyzing social media comments across three major Chinese platforms, applying both sentiment and content analysis. We extend prior literature by capturing a wider range of emotions, identifying their implications for health communication, and emphasizing how nurses can respond to the public’s emotional needs.

## 2. Method

### 2.1. Data collection

**Fig 1:**
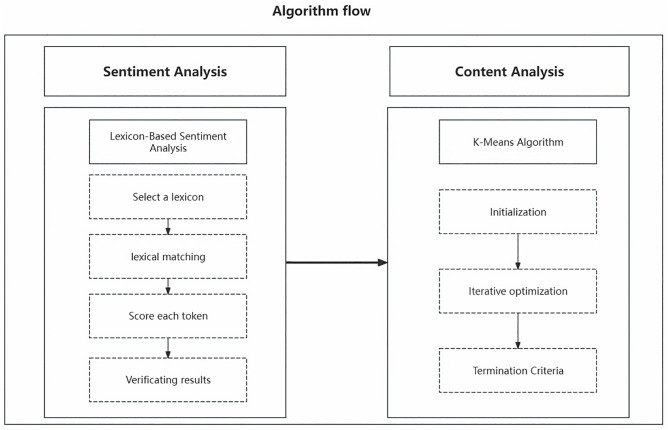
Algorithm flow.

Comments were collected from Zhihu (https://www.zhihu.com/), Xiaohongshu (https://www.xiaohongshu.com), and Weibo (https://www.weibo.com) from 2012 to August 10, 2024, using keywords such as “screening,” “mass screening,” “cancer screening,” and “secondary prevention.” All available comments matching these criteria during the timeframe were included, resulting in 796 comments. According to King’s recommendations [10], the user type includes non-healthcare, health professionals, media organizations, and unknown. Non-healthcare includes Self, family/friends, celebrities, and well-known. This step is implemented using Python’s Selenium library. Those steps are shown in Fig 1

### 2.2. Data cleaning

Chinese text preprocessing was performed using the jieba segmentation library. Stop words were removed using a Chinese stop-word dictionary, and synonyms were unified to improve consistency. This step is implemented using Python’s Numpy library. 1. Remove noisy data: Clean up non-text elements like HTML tags, emoticons, and URLs to reduce the irrelevant content. 2. Standardisation: Converting standard forms, such as symbols, spaces, and abbreviations, without changing the meaning of the text. 3. Spell Checker: The spell checker helps to quickly identify spelling mistakes in the text, increasing the speed and accuracy of the algorithm. 4. Remove unused words: Remove common words unrelated to sentiment analysis, such as ‘of,’ ‘is, “in,’ etc.

### 2.3. Data standardization

Data standardization improves the accuracy of sentiment analysis and content analysis. This step is implemented using the TfidfVectorizer of Python’s Sklearn library. 1. Segmentation: Simplifying critical components of a sentence improves the algorithm’s understanding of the sentence. 2. Feature extraction: textual data is transformed into numerical feature vectors. 3. Constructing the matrix: The TF-IDF document-word matrix represents the text as the number of word occurrences. Each matrix element represents the TF-IDF weight of the word in the text. 4. Normalisation: ensures that all text has the same scale, eliminating the effect of text length.

### 2.4. Sentiment analysis

1. Select a lexicon: We used Dalian University of Technology’s emotion dictionary, which contains word lexical categories, emotion categories, emotion intensity, and polarity. Using Ekman’s emotion classification approach, the lexicon has been updated with Chinese adaptations [24–26]. 2. Lexical matching: The cleaned text matches the sentiment lexicon. Second, the emotion words in the text are identified. Finally, the words are labeled positive, negative, or neutral emotions. 3. Score each token: Using weighting and counting methods to score the emotion on the labeled emotion words. 4. Verification results: This study used a two-person manual labeling comparison to ensure the accuracy of sentiment analysis. Two independent coders manually labeled a random subset of 100 comments. Inter-rater reliability was high (Cohen’s Kappa = 0.82), ensuring consistency.

### 2.5. Content analysis

We used the K-Means Algorithm for content analysis to obtain comprehensive and insightful results based on the sentiment analysis results. K-Means Algorithm is an unsupervised machine-learning method that explores potential patterns or themes in textual data [27]. Previous research by our research team has validated and optimized this algorithm in 4 steps. 1. Initialization: randomly select K clustering centers. 2. Iterative optimization: The distance from each data point to all cluster centers is calculated and assigned to the cluster with the closest distance. For each cluster, calculate the mean of all data points within the cluster and use the mean as the new cluster center. 3. Termination Criteria: The K clustering centers no longer changed significantly. The K value was determined using the elbow method, testing values from 3 to 10. A value of k = 7 was chosen as optimal, aligning with the seven emotion categories identified.

### 2.6. Ethics approval

Ethics approval was obtained from the Institutional Review Board of the Integrated Traditional and Western Medicine Hospital of Linping District (Approval No. 2024_09). All comments were anonymized, with usernames and identifiers removed. The study complied with Chinese data protection regulations. Data collection and analysis complied with the terms and conditions of each platform, including Zhihu, Xiaohongshu, and Weibo.

## 3. Results

This study included 796 comments, totaling 53,151 words. The results of the temporal analysis show that the increase in the level of discussion has been faster in the last three years, especially in 2024, when it is as high as 59.5 percent; May-July has the highest percentage (15.3%, 14.6%, 23.8%); and 9–15 clock has the highest percentage (6.6%, 6.6%, 6.0%, 5.6%, 7.6%, 8.3%, 6.3%). The results of user type showed that non-healthcare users comprised 68.6%, health professional(22.5%), media organization(6.5%), and unknown(2.4%).

The sentiment analysis results were classified into seven categories: good, happy, surprise, anger, disgust, fear, and sad, as shown in Table 1.

**Table 1:** The results of content analysis and sentiment analysis.

Attitude	Content Analysis Results	Sentiment Analysis Results
Good	Positive Psychological Drive	Health, Adherence, Good, Development, Guidance, Etc.
	Trust	Specialist, Hope, Positive, Breakthrough, Believe, Etc.
	Financial Support	Emphasis, Importance, Support, Compliance, Valuing, Etc.
	Screening Effectiveness	Effective, Accurate, Precise, Efficient, Breakthrough, Etc.
Happy	Resilience	Success, Celebration, Optimism, Progress, Fortunate, Etc.
	Safety	Reassurance, Enhance, Soothing, Successful, Peaceful, Etc.
Surprise	Self-Doubt	Strange, Magic, Surprising, Rare, Mysterious, Etc.
Anger	Impaired Self-Esteem	Outbursts, Humiliations,Temperament,Roar, Fires, Etc.
Disgust	Stigma and Embarrassment	Helpless, Invasive, Apathetic, Jerk, Rude
	Perceived Self-Vulnerability	Worried, Serious, Malignant, Suspicious, Nervous, Etc.
Fear	Lack of Knowledge	Terrifying, Virus, Fatal, Latent, Complications, Etc.
	Risk Perception	Fear, Scorn, Nightmare, Horror, Grim, Etc.
Sad	Procrastination and Low Perceived Susceptibility	Regrets, Remember, Inflammation, Information, Sorry, Etc.
	Regret of Anticipated Death	Pain, Loss, Homelessness, Dilemma, Divorce, Etc.
	Loss	Nothingness, Frustration, Helplessness, Despair, Death, Etc.
	Loneliness and Trauma	Loneliness, Trauma, Heartache, Grief, Funeral, Etc.

**Fig 2:**
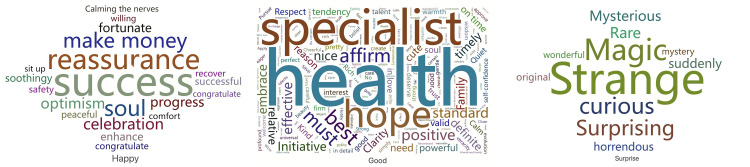
Positive emotions.

Positive emotions are shown in Fig 2. The results of positive emotions are divided into four categories. Words like health and persistence are linked to positive psychological drive. Words like Specialist and hope are linked to trust. Words like compliance and importance are linked to financial support. Words like accurate and compelling are linked to screening effectiveness. The results of happy emotions are divided into two categories. Words like success and celebration are linked to resilience. Words like reassurance and enhancement are linked to safety. The results of surprise emotions showed that Words like strange and magical are linked to self-doubt.

**Fig 3:**
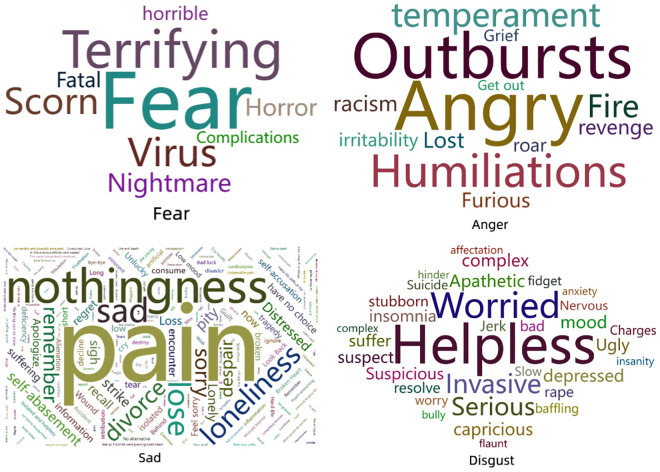
Negative emotions.

Negative emotions are shown in Fig 3. The results of anger emotions showed that Words like outbursts and humiliations are linked to impaired self-esteem. The results of disgust emotions are divided into two categories. Words like helpless and invasive are linked to stigma and embarrassment. Words like worried and severe are linked to perceived self-vulnerability. The results of fear emotions are divided into two categories. Words like terrifying and fatal are linked to a lack of Knowledge. Words like fear and nightmare are linked to risk perception. The results of sad emotions are divided into four categories. Words like regrets and remember are linked to procrastination and low perceived susceptibility. Words like pain, loss, and homelessness are linked to regret or anticipated death. Words like nothingness and frustration are linked to loss. Words like loneliness and trauma are linked to loneliness and trauma.

## 4. Discussion

This study revealed seven emotional categories related to cancer screening. Positive emotions highlight opportunities to build trust and resilience, while negative emotions reveal barriers such as fear, stigma, and procrastination. Condensing overlapping explanations avoids redundancy and emphasizes key contrasts between emotions.

Screeners experiencing positive emotions often described a sense of social obligation and responsibility, aligning with literature that emphasizes health behaviors as a form of civic participation [28–30]. Screening not only enhanced their personal well-being but also provided social recognition. Trust was another critical dimension: confidence in physicians and screening technologies directly reinforced belief in the process and encouraged participation [31–34]. In addition, participants emphasized the role of policy and financial support in reducing economic barriers, a finding consistent with prior research [35,36]. Lastly, the perceived effectiveness of screening—its accuracy and personalization—was a strong motivator, reducing misdiagnosis fears and fostering security [37–39]. Collectively, these aspects suggest that healthcare systems should emphasize social value, transparent communication, and technical quality, while governments should expand subsidies and funding to guarantee equitable access.

Happy emotion was expressed through resilience and safety. For some, screening represented an opportunity for early detection and reassurance of health, enabling them to confront illness with confidence [40,41]. Others emphasized a release of health concerns and confirmation of their preventive measures, leading to psychological well-being and greater engagement in healthy behaviors [33,42,43]. These findings suggest that professional counseling and consistent feedback from healthcare providers can reinforce resilience and provide reassurance, thereby sustaining long-term screening participation [43].

Unexpected screening results produced feelings of self-doubt and cognitive dissonance. Individuals who previously considered themselves healthy struggled to reconcile their self-image with the new information, which sometimes extended to skepticism toward the healthcare system [43,44]. Such experiences illustrate the fragile balance between information disclosure and patient confidence. While surprising results may catalyze reflection and behavior change, they may also provoke denial or avoidance. Addressing this requires sensitive communication strategies that normalize unexpected outcomes while guiding individuals toward constructive coping [45].

Anger emotions. Anger often stemmed from negative screening experiences or frustration at perceived loss of autonomy. Screeners who resisted acknowledging health problems sometimes redirected their anger toward healthcare providers or institutions [33,40,42]. If unmanaged, such emotions can erode trust in the system and discourage follow-up. Interventions should therefore focus on transparent communication, patient autonomy, and timely explanations of results. By fostering mutual respect and clear dialogue, healthcare professionals can transform anger into opportunities for improved patient engagement [46].

Disgust emotion was primarily linked to stigma, embarrassment, and loss of bodily autonomy during invasive procedures. Feelings of vulnerability intensified when individuals perceived a lack of privacy or control over their health [33,42,47]. These reactions highlight the importance of developing less invasive, more comfortable technologies and reducing the psychological burden of screening [48,49]. Additionally, education on the medical necessity of certain procedures, coupled with empathy-based care, can mitigate stigma and embarrassment, encouraging more open participation.

Fear emotions as a particularly complex and ambivalent response. On one hand, perceived risk motivated some to seek reassurance through screening; on the other, fear of diagnosis or treatment deterred participation [42,50,51]. Moreover, fear often extended beyond personal health to concerns about family responsibilities, stability, and social roles. Such dual effects confirm prior findings that fear can both mobilize and paralyze health behaviors [51–53]. Addressing this requires nuanced strategies: campaigns that clarify risks, decision-support tools that reduce uncertainty, and psychological therapies (e.g., cognitive behavioral approaches) to help individuals regulate fear without avoidance [54].

Sad emotion was the most multifaceted negative emotion, encompassing procrastination, regret, loss, anticipated death, and loneliness. Many screeners expressed grief for delaying screening, missing opportunities for early treatment, and fearing the worsening of illness [50,51,55]. Others described helplessness, mourning, and isolation, especially in the absence of social support [33,51,53]. Such findings emphasize the need for comprehensive psychosocial care that integrates emotional support, family involvement, and palliative planning when necessary. Healthcare professionals should actively encourage early screening, share success stories, and ensure continuity of care by coordinating with specialized services and support networks [23,44,54].

There are four limitations to this study. First, the Internet’s health professionals and media organizations represent a small part and may not reflect their attitudes. Therefore, we recommend that future studies use medical forum data and news media to supplement them. Second, social media platforms may amplify extreme emotions through algorithmic recommendations, which could bias the observed distribution. Moreover, our sample overrepresents younger users, while older adults are underrepresented. Third, emotions such as trust and fear were associated with reported screening attitudes, but causality cannot be established from observational data. Fourth, the Non-healthcare group has a large younger demographic but lacks an elderly demographic. Older persons are the main target population for cancer screening, but social media overlook their voices. Future studies should focus on older persons’ attitudes about cancer screening.

## 5. Conclusion

This study identified seven categories of emotions in Chinese social media discourse on cancer screening. Positive emotions can be leveraged to strengthen trust and participation, while negative emotions highlight psychological and social barriers. These insights provide guidance for designing emotionally responsive and culturally tailored nursing interventions. Specifically, nurses and health communicators should address fear and stigma through targeted education and counseling while reinforcing positive psychological drivers to promote early screening participation.

## References

[1] International Agency for Research on Cancer. Cancer fact sheets: All cancers. https://gco.iarc.fr/today/en. 2019. Accessed 2024 August 21.

[2] Chan DNS, So WKW. Effectiveness of motivational interviewing in enhancing cancer screening uptake amongst average-risk individuals: A systematic review. Int J Nurs Stud. 2021;113:103786. doi: 10.1016/j.ijnurstu.2020.103786 33091749

[3] Smith RA, Andrews KS, Brooks D, Fedewa SA, Manassaram-Baptiste D, Saslow D, et al. Cancer screening in the United States, 2019: A review of current American Cancer Society guidelines and current issues in cancer screening. CA Cancer J Clin. 2019;69(3):184–210. doi: 10.3322/caac.21557 30875085

[4] Chan DNS, So WKW. A Systematic Review of the Factors Influencing Ethnic Minority Women’s Cervical Cancer Screening Behavior: From Intrapersonal to Policy Level. Cancer Nurs. 2017;40(6):E1–30. doi: 10.1097/NCC.0000000000000436 28081032

[5] So WKW, Wong CL, Chow KM, Chen JMT, Lam WWT, Chan CWH, et al. The uptake of cervical cancer screening among South Asians and the general population in Hong Kong: A comparative study. Journal of Cancer Policy. 2017;12:90–6. doi: 10.1016/j.jcpo.2017.03.015

[6] Hamashima C, Saito H, Sobue T. Awareness of and adherence to cancer screening guidelines among health professionals in Japan. Cancer Sci. 2007;98(8):1241–7. doi: 10.1111/j.1349-7006.2007.00512.x 17537173 PMC11159036

[7] Heisler Z, Eastwood B, Mwaiselage J, Kahesa C, Msami K, Soliman AS. Return on Investment of a Breast Cancer Screening Program in Tanzania: Opportunity for Patient and Public Education. J Cancer Educ. 2022;37(3):701–8. doi: 10.1007/s13187-020-01871-6 32980979 PMC7997813

[8] Yu Z, Li B, Zhao S, Du J, Zhang Y, Liu X, et al. Uptake and detection rate of colorectal cancer screening with colonoscopy in China: A population-based, prospective cohort study. Int J Nurs Stud. 2024;153:104728. doi: 10.1016/j.ijnurstu.2024.104728 38461798

[9] Afroze T, Iyer A, Faisal H, Manaf H, Bahul R. Knowledge and Attitudes Regarding Breast Cancer Screening and Mammograms Among Women Aged 40 Years and Older in the United Arab Emirates. Cureus. 2024;16(5):e59766. doi: 10.7759/cureus.59766 38846223 PMC11153839

[10] King AJ, Margolin D, Tong C, Chunara R, Niederdeppe J. Making Sense of Social Media Data About Colorectal Cancer Screening. J Am Coll Radiol. 2024;21(4):543–4. doi: 10.1016/j.jacr.2023.06.045 37838186 PMC10954397

[11] Zubiaga A, Rosso P. Special issue on analysis and mining of social media data. PeerJ Comput Sci. 2024;10:e1909. doi: 10.7717/peerj-cs.1909 38435569 PMC10909232

[12] Li C, Liu Y, Xue D, Chan CWH. Effects of nurse-led interventions on early detection of cancer: A systematic review and meta-analysis. Int J Nurs Stud. 2020;110:103684. doi: 10.1016/j.ijnurstu.2020.103684 32702568

[13] Plackett R, Kaushal A, Kassianos AP, Cross A, Lewins D, Sheringham J, et al. Use of Social Media to Promote Cancer Screening and Early Diagnosis: Scoping Review. J Med Internet Res. 2020;22(11):e21582. doi: 10.2196/21582 33164907 PMC7683249

[14] Cutrona SL, Roblin DW, Wagner JL, Gaglio B, Williams AE, Torres Stone R, et al. Adult Willingness to Use Email and Social Media for Peer-to-Peer Cancer Screening Communication: Quantitative Interview Study. JMIR Res Protoc. 2013;2(2):e52. doi: 10.2196/resprot.2886 24287495 PMC3868965

[15] Zhao F, Yan H, Liu Y, Mu X, Wang D, Du J, et al. Knowledge, attitudes and practices toward cervical cancer screening among ethnic minorities in inner Mongolia, China. J Eval Clin Pract. 2024;30(8):1629–35. doi: 10.1111/jep.14074 38978407

[16] Döbrössy B, Girasek E, Susánszky A, Koncz Z, Győrffy Z, Bognár VK. “Clicks, likes, shares and comments” a systematic review of breast cancer screening discourse in social media. PLoS One. 2020;15(4):e0231422. doi: 10.1371/journal.pone.0231422 32294139 PMC7159232

[17] Elmaghraby DA, Alshalla AA, Alyahyan A, Altaweel M, Al Ben Hamad AM, Alhunfoosh KM, et al. Public Knowledge, Practice, and Attitude Regarding Cancer Screening: A Community-Based Study in Saudi Arabia. Int J Environ Res Public Health. 2023;20(2):1114. doi: 10.3390/ijerph20021114 36673870 PMC9859105

[18] Humaid Al-Shamsi S, Humaid Al-Shamsi A, Humaid Al-Shamsi M, Sajwani A, Alzaabi MS, Al Hammadi O, et al. The Perception and Awareness of the Public about Cancer and Cancer Screening in the United Arab Emirates, a Population-Based Survey. Clin Pract. 2023;13(3):701–14. doi: 10.3390/clinpract13030064 37366933 PMC10297532

[19] López-Panisello MB, Pérez-Lacasta MJ, Rué M, Carles-Lavila M. Factors influencing intention to participate in breast cancer screening. An exploratory structural model. PLoS One. 2023;18(2):e0281454. doi: 10.1371/journal.pone.0281454 36735750 PMC9897558

[20] Wu Z, Liu Y, Li X, Song B, Ni C, Lin F. Factors associated with breast cancer screening participation among women in mainland China: a systematic review. BMJ Open. 2019;9(8):e028705. doi: 10.1136/bmjopen-2018-028705 31455705 PMC6720337

[21] Chen JC, LeBedis CA, Chang KJ. The Public Perception of CT Colonography Versus Colonoscopy via Sentiment Analysis of Social Media. J Am Coll Radiol. 2023;20(6):531–6. doi: 10.1016/j.jacr.2023.03.011 37127218

[22] Metwally O, Blumberg S, Ladabaum U, Sinha SR. Using Social Media to Characterize Public Sentiment Toward Medical Interventions Commonly Used for Cancer Screening: An Observational Study. J Med Internet Res. 2017;19(6):e200. doi: 10.2196/jmir.7485 28592395 PMC5480009

[23] Nastasi A, Bryant T, Canner JK, Dredze M, Camp MS, Nagarajan N. Breast Cancer Screening and Social Media: a Content Analysis of Evidence Use and Guideline Opinions on Twitter. J Cancer Educ. 2018;33(3):695–702. doi: 10.1007/s13187-017-1168-9 28097527

[24] Cero I, Luo J, Falligant JM. Lexicon-Based Sentiment Analysis in Behavioral Research. Perspect Behav Sci. 2024;47(1):283–310. doi: 10.1007/s40614-023-00394-x 38660506 PMC11035532

[25] Yazdani A, Shamloo M, Khaki M, Nahvijou A. Use of sentiment analysis for capturing hospitalized cancer patients’ experience from free-text comments in the Persian language. BMC Med Inform Decis Mak. 2023;23(1):275. doi: 10.1186/s12911-023-02358-2 38031102 PMC10685532

[26] Zhou Q, Xu Y, Yang L, Menhas R. Attitudes of the public and medical professionals toward nurse prescribing: A text-mining study based on social medias. Int J Nurs Sci. 2023;11(1):99–105. doi: 10.1016/j.ijnss.2023.12.005 38352288 PMC10859581

[27] Zhou Q, Lei Y, Du H, Tao Y. Public concerns and attitudes towards autism on Chinese social media based on K-means algorithm. Sci Rep. 2023;13(1):15173. doi: 10.1038/s41598-023-42396-4 37704712 PMC10499991

[28] Adsul P, Nayaka S, Pramathesh R, Gowda S, Jaykrishna P, Srinivas V, et al. Using photovoice to understand the context of cervical cancer screening for underserved communities in rural India. Glob Health Promot. 2020;27(4):50–8. doi: 10.1177/1757975920915677 32400290 PMC7666022

[29] Monu JI, Achar C, Wood DE, Flum DR, Agrawal N, Farjah F. Psychological Traits and the Persuasiveness of Lung Cancer Screening Health Messages. Ann Thorac Surg. 2022;113(4):1341–7. doi: 10.1016/j.athoracsur.2021.04.047 33957098 PMC8563489

[30] Tribe C, Webb J. Avoiding piecemeal research on participation in cervical cancer screening: the advantages of a social identity framework. Health Expect. 2014;17(4):453–65. doi: 10.1111/j.1369-7625.2012.00779.x 22646802 PMC5060747

[31] Golden SE, Ono SS, Thakurta SG, Wiener RS, Iaccarino JM, Melzer AC, et al. “I’m Putting My Trust in Their Hands”: A Qualitative Study of Patients’ Views on Clinician Initial Communication About Lung Cancer Screening. Chest. 2020;158(3):1260–7. doi: 10.1016/j.chest.2020.02.072 32278782

[32] Hong HC, Ferrans CE, Park C, Lee H, Quinn L, Collins EG. Effects of Perceived Discrimination and Trust on Breast Cancer Screening among Korean American Women. Womens Health Issues. 2018;28(2):188–96. doi: 10.1016/j.whi.2017.11.001 29223326

[33] James LJ, Wong G, Craig JC, Hanson CS, Ju A, Howard K, et al. Men’s perspectives of prostate cancer screening: A systematic review of qualitative studies. PLoS One. 2017;12(11):e0188258. doi: 10.1371/journal.pone.0188258 29182649 PMC5705146

[34] O’Donovan B, Mooney T, Rimmer B, Fitzpatrick P, Flannelly G, Doherty L, et al. Trust and cancer screening: Effects of a screening controversy on women’s perceptions of cervical cancer screening. Prev Med Rep. 2021;25:101684. doi: 10.1016/j.pmedr.2021.101684 35127361 PMC8800010

[35] Nguyen TXT, Lal S, Abdul-Salam S, Khan MSR, Kadoya Y. Financial Literacy, Financial Education, and Cancer Screening Behavior: Evidence from Japan. Int J Environ Res Public Health. 2022;19(8):4457. doi: 10.3390/ijerph19084457 35457329 PMC9030491

[36] Jones SMW, Schuler TA, Padamsee TJ, Andersen MR. Financial Anxiety is Associated With Cancer Screening Adherence in Women at High Risk of Breast Cancer. Ann Behav Med. 2021;55(12):1241–5. doi: 10.1093/abm/kaab010 33761532

[37] Abramson M, Feiertag N, Javidi D, Babar M, Loeb S, Watts K. Accuracy of prostate cancer screening recommendations for high-risk populations on YouTube and TikTok. BJUI Compass. 2022;4(2):206–13. doi: 10.1002/bco2.200 36816146 PMC9931542

[38] Chubak J, Burnett-Hartman AN, Barlow WE, Corley DA, Croswell JM, Neslund-Dudas C, et al. Estimating Cancer Screening Sensitivity and Specificity Using Healthcare Utilization Data: Defining the Accuracy Assessment Interval. Cancer Epidemiol Biomarkers Prev. 2022;31(8):1517–20. doi: 10.1158/1055-9965.EPI-22-0232 35916602 PMC9484579

[39] Roberts MC, Ferrer RA, Rendle KA, Kobrin SC, Taplin SH, Hesse BW, et al. Lay Beliefs About the Accuracy and Value of Cancer Screening. Am J Prev Med. 2018;54(5):699–703. doi: 10.1016/j.amepre.2018.02.002 29551327 PMC5911403

[40] Hajek A, Bock J-O, König H-H. The role of general psychosocial factors for the use of cancer screening-Findings of a population-based observational study among older adults in Germany. Cancer Med. 2017;6(12):3025–39. doi: 10.1002/cam4.1226 29030910 PMC5727314

[41] Wang G, Li Z, Luo X, Wei R, Liu H, Yang J, et al. Effects of nurse-led supportive-expressive group intervention for post-traumatic growth among breast cancer survivors: A randomized clinical trial. J Nurs Scholarsh. 2022;54(4):434–44. doi: 10.1111/jnu.12752 34898001

[42] Agurto I, Bishop A, Sánchez G, Betancourt Z, Robles S. Perceived barriers and benefits to cervical cancer screening in Latin America. Prev Med. 2004;39(1):91–8. doi: 10.1016/j.ypmed.2004.03.040 15207990

[43] Health Quality Ontario. Women’s Experiences of Inaccurate Breast Cancer Screening Results: A Systematic Review and Qualitative Meta-synthesis. Ont Health Technol Assess Ser. 2016;16(16):1–22. 27468327 PMC4947976

[44] Fong J, Venables M, D’Souza D, Maskerine C. Patient Communication Preferences for Prostate Cancer Screening Discussions: A Scoping Review. Ann Fam Med. 2023;21(5):448–55. doi: 10.1370/afm.3011 37748915 PMC10519764

[45] Wood LD, Canto MI, Jaffee EM, Simeone DM. Pancreatic Cancer: Pathogenesis, Screening, Diagnosis, and Treatment. Gastroenterology. 2022;163(2):386-402.e1. doi: 10.1053/j.gastro.2022.03.056 35398344 PMC9516440

[46] Estevan-Vilar M, Parker LA, Caballero-Romeu JP, Ronda E, Hernández-Aguado I, Lumbreras B. Barriers and facilitators of shared decision-making in prostate cancer screening in primary care: A systematic review. Prev Med Rep. 2023;37:102539. doi: 10.1016/j.pmedr.2023.102539 38179441 PMC10764268

[47] Choi EPH, Wan EYF. Attitude Toward Prostate Cancer Screening in Hong Kong: The Importance of Perceived Consequence and Anticipated Regret. Am J Mens Health. 2021;15(5). doi: 10.1177/15579883211051442 34622702 PMC8504245

[48] Le Bonniec A, Meade O, Fredrix M, Morrissey E, O’Carroll RE, Murphy PJ, et al. Exploring non-participation in colorectal cancer screening: A systematic review of qualitative studies. Soc Sci Med. 2023;329:116022. doi: 10.1016/j.socscimed.2023.116022 37348182

[49] Opondo CO, Onyango PO, Asweto CO. Effect of Perceived Self-Vulnerability on Prostate Cancer Screening Uptake and Associated Factors: A Cross-Sectional Study of Public Health Facilities in Western Kenya. Ann Glob Health. 2022;88(1):12. doi: 10.5334/aogh.3064 35281883 PMC8855733

[50] Alam Z, Shafiee Hanjani L, Dean J, Janda M. Cervical Cancer Screening Among Immigrant Women Residing in Australia: A Systematic Review. Asia Pac J Public Health. 2021;33(8):816–27. doi: 10.1177/10105395211006600 33829888

[51] Azami-Aghdash S, Ghojazadeh M, Sheyklo SG, Daemi A, Kolahdouzan K, Mohseni M, et al. Breast Cancer Screening Barriers from the Womans Perspective: a Meta-synthesis. Asian Pac J Cancer Prev. 2015;16(8):3463–71. doi: 10.7314/apjcp.2015.16.8.3463 25921163

[52] Honein-AbouHaidar GN, Kastner M, Vuong V, Perrier L, Daly C, Rabeneck L, et al. Systematic Review and Meta-study Synthesis of Qualitative Studies Evaluating Facilitators and Barriers to Participation in Colorectal Cancer Screening. Cancer Epidemiol Biomarkers Prev. 2016;25(6):907–17. doi: 10.1158/1055-9965.EPI-15-0990 27197277

[53] Kasraeian M, Hessami K, Vafaei H, Asadi N, Foroughinia L, Roozmeh S, et al. Patients’ self-reported factors influencing cervical cancer screening uptake among HIV-positive women in low- and middle-income countries: An integrative review. Gynecol Oncol Rep. 2020;33:100596. doi: 10.1016/j.gore.2020.100596 32551354 PMC7292910

[54] Saraiya M, Colbert J, Bhat GL, Almonte R, Winters DW, Sebastian S, et al. Computable Guidelines and Clinical Decision Support for Cervical Cancer Screening and Management to Improve Outcomes and Health Equity. J Womens Health (Larchmt). 2022;31(4):462–8. doi: 10.1089/jwh.2022.0100 35467443 PMC9206487

[55] Rajendram P, Singh P, Han KT, Utravathy V, Wee HL, Jha A, et al. Barriers to breast cancer screening in Singapore: A literature review. Ann Acad Med Singap. 2022;51(8):493–501. doi: 10.47102/annals-acadmedsg.2021329 36047524

